# Design and Implementation of a Nutrition and Physical Activity Curriculum for Child Care Settings

**Published:** 2006-03-15

**Authors:** Carolyn Dunn, Cathy Thomas, Dianne Ward, Kelly Webber, Courtney Cullitan, Leslie Pegram, Kelly Webber

**Affiliations:** North Carolina State University, Department of Family and Consumer Sciences, North Carolina Cooperative Extension; North Carolina Division of Public Health, Raleigh, NC; Department of Nutrition, University of North Carolina at Chapel Hill, Chapel Hill, NC; Department of Nutrition, University of North Carolina at Chapel Hill, Chapel Hill, NC; Department of Nutrition, University of North Carolina at Chapel Hill, Chapel Hill, NC. Ms Cullitan is now a Child Nutrition Manager with East Coast Migrant Head Start, Raleigh, NC; North Carolina Cooperative Extension, Raleigh, NC; Department of Nutrition, University of North Carolina at Chapel Hill, Chapel Hill, NC

## Abstract

**Background:**

Childhood overweight continues to increase in the United States. Children should begin establishing healthy eating and physical activity behaviors at a young age.

**Context:**

Many children spend a large part of their day in child care settings, whether in preschools or home day care settings. Child care providers in these settings have an opportunity to establish and reinforce habits that promote good health. However, the providers need training and creative educational materials to teach children about healthy eating and physical activity. *Color Me Healthy* is an educational program focusing on nutrition and physical activity that was developed for children aged 4 and 5 years by three of the authors (C.D., C.T., and L.P.).

**Methods:**

In 2001 and 2002, the program was implemented in 47 North Carolina counties and the North Carolina Cherokee reservation. In December 2001, we used an information-dissemination model called Train the Trainer during a session to teach county teams comprising local public health professionals and cooperative extension employees how to teach child care providers in their communities to use the curriculum. The child care providers were then trained between March and August 2002. Follow-up evaluation forms were given to trained child care providers 8 weeks after the training.

**Consequences:**

Of the providers who completed the evaluations (n = 486), 92.0% indicated that using the *Color Me Healthy* curriculum increased the physical activity of their students, and 91.8% indicated that it increased the children's knowledge about movement. In addition, 93.0% of providers also indicated that using *Color Me Healthy* had increased the children's knowledge about healthy eating.

**Interpretation:**

Child care providers need educational materials on healthy eating and physical activity and should be trained to use them. The Train the Trainer model is an effective way to teach public health professionals to train child care providers on the *Color Me Healthy* curriculum materials about healthy eating and physical activity.

## Background

Childhood overweight is increasing in the United States. Currently, one in five children is overweight or at risk of becoming overweight (1). In the United States, the prevalence of childhood overweight tripled between 1980 and 2000 (2). Specifically, 10.4% of children aged 2 to 5 years are overweight (≥95th percentile body mass index [BMI] for age), and 20.6% are at risk of overweight (85th to < 95th percentile BMI for age) (3). The prevalence of overweight in this age group is even higher among Mexican Americans, with 11.1% being overweight and 22.7% being at risk of overweight (3). The epidemic of childhood overweight is a complex problem with many contributing factors. Lack of adequate physical activity and unhealthy eating habits are widely recognized issues; however, very few children have healthy eating and physical activity habits that would decrease their risk of overweight (4,5).

Many children's eating patterns are not consistent with current recommendations for a healthy diet (4,5). Children consume too much fat and sugar-sweetened beverages and too little fiber, fruit, and vegetables (2,6). In addition, lack of physical activity is associated with higher weights in children. It is recommended that children participate in at least 60 minutes of physical activity per day, but many children do not meet this recommendation (5,7).

## Context

Every day, more than 13 million preschool-age (3 to 5 years) children are in child care settings such as preschools or home day care settings, which could present excellent opportunities for the child care providers to reinforce healthy eating and physical activity habits. It is imperative that at a young age, children are taught about healthy eating and the benefits of physical activity (8). Furthermore, children may then be able to influence the health behaviors of their own families (9,10).

Involving caregivers in educating and inspiring children about healthy eating and physical activity is an important way adults can help children develop behaviors that can prevent overweight. However, many child care providers lack training in nutrition and physical activity education.

The purpose of this article is to describe the development and initial implementation of *Color Me Healthy, *a healthy eating and physical activity program for children aged 4 and 5 years in child care settings.

Health promotion should include multiple strategies including education, advocacy, organizational change, policy change, and environmental change, emphasizing a complete approach involving the individual, family, and community. *Color Me Healthy* is a curriculum that was developed based on this philosophy (11,12). Social cognitive theory (13) and the socioecological model (12) were used to guide program development. Social cognitive theory explains the way people acquire and maintain their behavior patterns and provides the basis for intervention strategies; changing behavior involves the environment, people, and the behavior itself. The socioecological model is also used as a framework for designing and implementing health education programs and is based on the idea that five levels of influence on health and health behavior exist: individual, interpersonal, organizational, community, and society. The model suggests that humans are shaped by their environments, which comprise many settings. According to the socioecological model, interventions are more effective if they address all five levels of influence; therefore, the *Color Me Healthy* curriculum components address all five levels (Table 1).

## Methods

### Curriculum development and components

*Color Me Healthy* was developed by three of the authors (C.D., C.T., and L.P.) and is a program designed to increase physical activity and promote healthy eating among children aged 4 and 5 years. An advisory committee of subject matter experts, child development professionals, and child care professionals reviewed all of the curriculum materials.

*Color Me Healthy* incorporates color, music, and the senses to teach children that healthy food and physical activity are fun. All materials needed to implement the *Color Me Healthy* program are provided in the curriculum kit, which includes the following:

**1. Teacher's guide:** The *Color Me Healthy* teacher's guide contains 12 Circle Time lesson plans. Each lesson contains the purpose of the lesson, a list of materials needed, and specific steps to carry out the lesson. The guide also contains the Color You Active section, which includes descriptions of six trips that allow the children to use their imagination to "travel" to different places and events. The teacher reads a story about visiting a destination, and the children act out events along the way. The Color Your Classroom section of the guide provides bulletin board and display suggestions. Because teachers are role models for children, the guide also includes Color You Healthy, a section that provides strategies for ways teachers can eat healthy and stay active.

**2. Picture cards:** The *Color Me Healthy* kit includes four sets of picture cards that are used in many of the Circle Time lessons. Colors of Foods includes eight cards with a color on one side and fruits and vegetables of the same color on the other side. Where the Foods Grow includes 15 cards with a single fruit or vegetable on one side and a description of where it grows on the other side. Places to Be Active has five cards with pictures of either a park, a backyard, the beach, the mountains, or a swimming pool on one side and activities children can do in these settings on the other side. The Dairy Foods card shows children all the foods that come from milk.

**3. Classroom posters:** Posters are used as educational tools in many of the Circle Time lessons. Three classroom posters are included in the kit: the *Color Me Healthy* logo poster, the Colors of Foods poster, and the Pretend You Are a . . . poster, which uses the alphabet to encourage children to be physically active.

**4. Music:** Music is used in many of the Circle Time lessons. Seven original songs were written to convey healthy eating and physical activity messages. Songs include the *Color Me Healthy Theme Song, Heartbeat Beat, Try New Foods, Play Outside, Taste the Colors, The Picnic Song,* and the *Color Me Healthy Dance Mix*.

**5. Hand stamp:** A self-inking hand stamp is included and can be used as a reward for participation.

**6. Parent component:** The kit includes a series of 14 reproducible newsletters that reinforce messages the children are learning in the classroom. In addition, two posters for parents are included that convey a basic message about healthy eating and physical activity that is similar to what the children are learning in the classroom. The parent posters are displayed in the child care setting in areas where parents typically collect information about their children.

### Train the Trainer model

In December 2001, *Color Me Healthy* county teams comprising family and statewide representatives from the North Carolina Cooperative Extension and representatives from the North Carolina Division of Public Health attended a 1-day Train the Trainer workshop to learn how to teach child care providers in their community about the program. During the workshop, teams received information on nutrition and physical activity for young children as well as materials needed to implement the program locally.

The training for county teams involved promotional strategies, such as visual displays of material, music, and singing, to promote interest and motivate the attendees. Team members modeled lessons and practiced techniques they could use to train child care providers. Each county team received a training manual that included a PowerPoint presentation, evaluation instruments, sample training agendas, and marketing materials. County teams received instructions on how to conduct local training sessions as well as all the educational materials needed to help promote consistency among all local training sessions.

Teams were instructed to develop a county *Color Me Healthy* dissemination plan for 2002. Training formats (4 hour, 1 day, or 2 day) and timing (typically nights or weekends) were selected based on local needs and preferences. Teams were encouraged to model their county-level child care provider trainings on the statewide training design and to use the sample agendas provided in their training manual. Child care providers received the *Color Me Healthy* kit after completing the training.

### Evaluation by child care providers

Child care providers who attended training during the first 6 months after the December 2001 Train the Trainer event (53 trainings with a total of 1338 participants) were given training evaluation forms immediately after their training session ended. Providers were asked to rate how effectively the training prepared them to implement the *Color Me Healthy* curriculum (with 94.4% reporting excellent or very good effectiveness) and to rate the *Color Me Healthy* materials (with 97.0% rating the materials as excellent or very good). In addition, providers were given an opportunity to give the trainers feedback on how to improve the training in the future. At the training session, child care providers were asked if they would be willing to provide a follow-up evaluation in 8 weeks*.*

The 38-item 8-week follow-up evaluation was designed and reviewed by subject matter specialists and education professionals and tested for readability by a small group of child care providers. Of the child care providers who attended training, 76.4% (1023) agreed to provide an 8-week follow-up evaluation. Participants were not asked to provide a reason for nonparticipation in the follow-up survey; however, many were not willing to provide their addresses or indicated that they would have to check with the owner of the child care center before agreeing to participate. Participants had the option of receiving their 8-week follow-up survey through the regular mail (899 participants) or e-mail (124 participants). Participants who did not respond to the 8-week follow-up evaluation received another evaluation 11 weeks after the training. Of the participants who initially agreed to complete the follow-up evaluation, 48.0% (486) participants completed and returned the survey, which was 36.3% of all individuals trained during the first 6 months of the program. (None of the surveys provided via e-mail were returned, indicating that e-mail may be an ineffective communication method for child care providers.)

Participants who completed the evaluation worked in child care centers (48.0%), home day care settings (34.7%), Head Start programs (7.9%), and other settings (9.5%); 74.0% participated in the Child and Adult Care Food Program. Six of the seven components provided in the *Color Me Healthy* curriculum were used by more than 85.0% of the respondents; 66.7% of respondents used the parent newsletters (Table 2).

Of the participating providers, 92.0% indicated that using *Color Me Healthy* increased the physical activity of the children in their care, and 91.8% indicated that it increased the children's knowledge about movement. In addition, 93.0% of providers indicated that using *Color Me Healthy* increased the children's knowledge about healthy eating. Of participating providers, 79.0% indicated that the children were more willing to try new foods, and 82.0% reported that the curriculum had improved fruit and vegetable recognition. The providers' responses were used to assess an increase in knowledge and behavior changes in the children. We hoped to assess the providers' perception of the curriculum's effectiveness because we knew it would affect future implementation.

Many providers (92.3%) indicated that using *Color Me Healthy* had helped them realize the importance of teaching children about nutrition. Most child care providers rated *Color Me Healthy* to be excellent (73.5%) or very good (23.6%); only 2.9% rated the program as good, and none rated it as fair or poor. Almost all the child care providers (99.8%) indicated they would use *Color Me Healthy* in the future.

## Consequences

Providing children with experiential learning in a fun environment has been recognized as a developmentally appropriate strategy for educating preschool children (14). *Color Me Healthy* is a preschool curriculum that addresses physical activity and healthy eating; it is designed to be teacher friendly, upbeat, and fun. Overall, the *Color Me Healthy* curriculum was positively received by child care providers, and they were using all the provided materials. We attribute the positive response to several factors.

First, the *Color Me Healthy* kit contains all the materials needed to implement the program. The kits were produced with the preschool classroom in mind and include a sturdy plastic case, laminated picture cards, a durable spiral-bound teacher's guide, and full-color materials.

Second, training sessions were provided for the trainers, who were then prepared to teach the child care providers. The Train the Trainer model is an effective way to disseminate educational programs (15). It allowed many counties to implement the *Color Me Healthy* program using existing county extension and public health personnel. In addition, the *Color Me Healthy* Train the Trainer workshops included hands-on learning experiences such as modeling Circle Time activities. The training motivated the county teams and prepared them to train child care providers in their communities. Because the teams received a training manual for use in provider training sessions, they were not required to develop any additional materials.

Child care providers received training from the county teams. Child care providers have limited time to prepare lesson plans; the trainings gave them the opportunity to become familiar with the curriculum materials and participate in lessons. In addition, the trainings encouraged child care providers to teach children about physical activity and healthy eating. Providing training to child care providers was essential for the implementation of the program in the classroom. Providers often lack formal training in nutrition and physical activity. Training for child care providers has been shown to be an effective method for improving the quality of care children receive and increasing their exposure to educational opportunities (16,17). Furthermore, using hands-on experiential methods during training is an effective method of ensuring that methods taught during training are implemented in the classroom (18).

Finally, the power of the state and county partnerships played a role. The partnership between cooperative extensions and the state division of public health provided a model for the counties. The state partnership benefited from combined financial and human resources, which ensured consistent physical activity and nutrition messages for the target population. In addition, the county teams had an existing relationship with child care providers and a history of training them. Working together as a team maximized the use of human resources without duplication of services. The use of county teams instead of single trainers provided program continuity even during county team staff turnover.

Of all the kit components, the parent newsletters were the least used. Parent newsletters were provided as paper documents that could be copied for distribution. The newsletter was the only component of the curriculum that the child care provider had to duplicate for distribution. Even though the cost would have been minimal, providers may not have had the resources, time, or motivation to copy the forms. If this were true, it would reinforce the importance of providing all needed components for the curriculum to increase the likelihood of use.

The training evaluation and 8-week follow-up evaluation were designed to accommodate providers with limited written communication skills and a low reading ability. Thus, the evaluation instruments did not ask for detailed or lengthy explanations. Feedback on the use and viability of the *Color Me Healthy* curriculum were provided by 486 (approximately 36%) of the child care providers who attended training in the first 6 months of the program. Of this group, an overwhelming number indicated that the materials were useful in educating the children about physical activity and healthy eating. It is not known whether the participants who were unwilling to participate in the 8-week follow-up evaluation (or who agreed to do so but did not complete the evaluation) had a similar opinion. Unfortunately, child care providers have little extra time during their day to complete evaluations, and job turnover is high.

## Interpretation

*Color Me Healthy* is a response to a need for nutrition and physical activity education materials in preschool settings. Child care providers need fun, innovative curriculum materials to address physical activity and healthy eating, but they often lack the formal training needed to use these materials without assistance. Using the Train the Trainer model is an effective way to train people to provide child care providers with information and curriculum materials on physical activity and healthy eating.

Partnerships between agencies at the state and county levels provide a rich infrastructure for the implementation and dissemination of successful programs. The county teams were able to successfully train child care providers in using the *Color Me Healthy* curriculum because of the training and materials they received at a statewide workshop. This allowed for consistency in training and messages delivered to child care providers across the state.

## Figures and Tables

**Table 1:** Socioecological Model Levels of Influence and Corresponding *Color Me Healthy* Components

**Level of Influence**	***Color Me Healthy* Component**

**Individual**	

Motivate change in individual behavior by increasing knowledge and influencing attitudes.	Educate children, child care providers, and parents.

**Interpersonal**	

Because groups provide social identity and support, interpersonal interventions target groups such as family members.	Target parents through two parent posters and *Color Me Healthy* newsletters. Teachers are role models for children. Children look to teachers for how to act, how to eat, and how to move. Thus, *Color Me Healthy* provides child care providers with information on physical activity and healthy eating in their own lives.

**Organizational**	

Change the policies, practices, and physical environment of an organization to support behavior change.	Training provides child care providers with the skills and resources to educate children about healthy eating and physical activity. *Color Me Healthy* influences the amount and quality of nutrition education and physical activity opportunities the children receive. *Color Me Healthy* provides child care providers with the tools to make their classroom a colorful, inviting environment, which is an important aspect of the learning process.

**Community**	

Coordinate the efforts of all members of a community to bring about change.	Parents are encouraged to examine their environment and assess the availability of physical activity and healthy eating options. Training for child care providers includes raising awareness about the importance of bike lanes, sidewalks, parks, and other opportunities to be physically active and eat healthy foods. Increasing awareness about the importance of building communities that provide opportunities to be active and eat healthy foods is critical to building and supporting healthy community environments.

**Society**	

Develop and enforce state policies and laws that can increase beneficial health behaviors.	*Color Me Healthy* is part of a larger statewide initiative that is working to influence policies and environments in support of physical activity and healthy eating. *Eat Smart, Move More . . . North Carolina* (www.eatsmartmovemorenc.com) is a statewide initiative that promotes increased opportunities for healthy eating and physical activity through policy and environmental changes.

**Table 2:** Results of 8-Week Follow-up Evaluation of *Color Me Healthy* (n = 486)

***Color Me Healthy* Curriculum Component**	**Respondents Who Used the Component,%**	**Of Respondents Who Used the Component**	

		**Respondents Who Liked the Component,%**	**Respondents Who Intend to Use the Component in the Future,%**

**Teacher's guide**			

Circle Time	94.0	99.1	99.4
Color Me Active imaginary trips	85.2	99.4	98.8
Color Your Classroom	84.8	97.1	97.2
*Color Me Healthy* song book	94.5	98.9	98.6
Color You Healthy	90.1	97.8	98.4
Resources	81.0	96.6	96.8

**Classroom posters**			

*Color Me Healthy* logo	77.3	95.0	95.8
Pretend You Are a . . .	83.4	97.1	98.1
Colors of Foods	94.3	98.9	98.9

**Parent posters**			

Remember to Eat All Your Colors	81.0	96.7	97.4
It All Counts	72.8	93.3	95.0

**Picture cards**			

Places to Be Active	91.1	99.1	99.1
Colors of Foods	97.1	99.4	99.4
Dairy Products	91.9	98.5	99.1
Where Foods Grow	93.3	98.6	98.6

**Music**			

CD	89.6	97.5	97.6
Cassette tape	80.3	92.4	92.4

**Parent newsletters**			

Welcome newsletter	68.7	94.0	92.9
Song lyric newsletter	66.1	92.0	91.6
Monthly newsletters	65.2	94.0	91.9

**Other**			

Hand stamp	84.5	97.5	97.6
